# Sulforaphane Analogues with Heterocyclic Moieties: Syntheses and Inhibitory Activities against Cancer Cell Lines

**DOI:** 10.3390/molecules21040514

**Published:** 2016-04-21

**Authors:** Ye-Hui Shi, Dong-Fang Dai, Jing Li, Yan-Wei Dong, Yin Jiang, Huan-Gong Li, Yuan Gao, Chuan-Ke Chong, Hui-Ying Li, Xiao-Qian Chu, Cheng Yang, Quan Zhang, Zhong-Sheng Tong, Cui-Gai Bai, Yue Chen

**Affiliations:** 1 Department of Breast Oncology, Tianjin Medical University Cancer Institute and Hospital, National Clinical Research Center for Cancer, Key Laboratory of Breast Cancer Prevention and Therapy, Tianjin Medical University, Ministry of Education, and Key Laboratory of Cancer Prevention and Therapy, Tianjin 30060, China; carfield@medmail.com.cn; 2 The State Key Laboratory of Elemento-Organic Chemistry, Collaborative Innovation Center of Chemical Science and Engineering (Tianjin), College of Pharmacy, and Tianjin Key Laboratory of Molecular Drug Research, Nankai University, Tianjin 300071, China; dongfangshion@163.com (D.-F.D.); zh116z@163.com (J.L.); ywdong_good@126.com (Y.-W.D.); jiangyin_001@163.com (Y.J.); 2120151007@mail.nankai.edu.cn (H.-G.L.); icanfly0@163.com (Y.G.); chongchuanke@mail.nankai.edu.cn (C.-K.C.); cyang66_2001@yahoo.com (C.Y.); zhangquan@nankai.edu.cn (Q.Z.); 3 High-throughput Molecular Drug Discovery Center, Tianjin International Joint Academy of BioMedicine, Tianjin 300457, China; lihuiying130892@163.com (H.-Y.L.); chuxiaoqian8@163.com (X.-Q.C.)

**Keywords:** sulforaphane (SFN), analogues, water soluble derivative, KG-1a, SUM-159, MCF-7, caspase-3, ALDH^+^

## Abstract

Recent studies have shown that sulforaphane (SFN) selectively inhibits the growth of ALDH^+^ breast cancer stem-like cells.Herein, a series of SFN analogues were synthesized and evaluated against breast cancer cell lines MCF-7 and SUM-159, and the leukemia stem cell-like cell line KG-1a. These SFN analogues were characterized by the replacement of the methyl group with heterocyclic moieties, and the replacement of the sulfoxide group with sulfide or sulfone. A growth inhibitory assay indicated that the tetrazole analogs **3d**, **8d** and **9d** were significantly more potent than SFN against the three cancer cell lines. Compound **14c**, the water soluble derivative of tetrazole sulfide **3d**, demonstrated higher potency against KG-1a cell line than **3d**. SFN, **3d** and **14c** significantly induced the activation of caspase-3, and reduced the ALDH^+^ subpopulation in the SUM159 cell line, while the marketed drug doxrubicin(DOX) increased the ALDH^+^ subpopulation.

## 1. Introduction

The natural compound, sulforaphane (SFN), was first isolated from broccoli in 1992. Since then, SFN has been found to be an effective chemo-preventive agent, and it exhibits anti-inflammatory, antioxidant, anti-proliferative and anti-cancer activities [1,2,3,4,5,6,7]. Recently, Sun *et al.* reported that SFN also inhibits the growth of the ALDH^+^ subpopulation of the breast cancer stem cell line, SUM-159, via down regulation of the Wnt/β-catenin self-renewal pathway [8]. Analogs of SFN were subsequently synthesized and their anti-cancer activities against various cancer cell lines were examined in the literature [1,4,9,10,11,12,13,14,15,16,17,18,19], and it was found that the replacement of the methyl group yielded compounds with significant activity [1,19]. Herein, a series of SFN analogues containing a heterocyclicring were synthesized, and then were evaluated for their activities against the breast cancer cell lines MCF-7 and SUM-159, and the leukemia stem cell-like KG-1a.

## 2. Results and Discussion

### 2.1. Synthesis of Sulforaphane Analogues and Water Soluble Derivatives

**Scheme 1:**
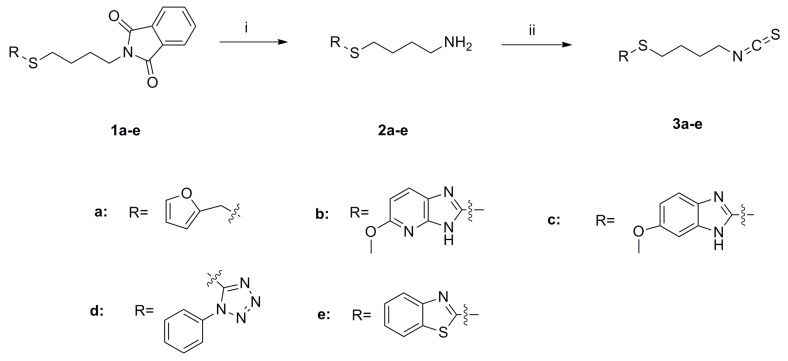
Synthesis of isothiocyanates **3a**–**e**. *Reagents and conditions*: (i) Me-NH_2_, MeOH, r.t., 70%–85% (ii) CSCl_2_, NaOH (aq, 1 mol/L), CH_2_Cl_2_, 0 °C to r.t., 37%–81%.

**Scheme 2:**
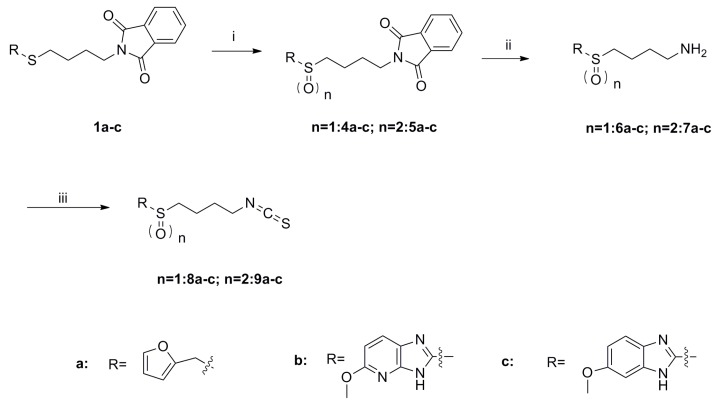
Synthesis of isothiocyanates **8a**–**c** and **9a**–**c**. *Reagents and conditions*: (i) TBHP, Ti(O-i-Pr)_4_, CH_2_Cl_2_, −20 to −10 °C, 58%–95%; (ii) Me-NH_2_, MeOH, r.t., 48%–64%; (iii) CSCl_2_, NaOH (aq, 1 mol/L), CH_2_Cl_2_, 0 °C to r.t., 53%–80%.

**Scheme 3:**
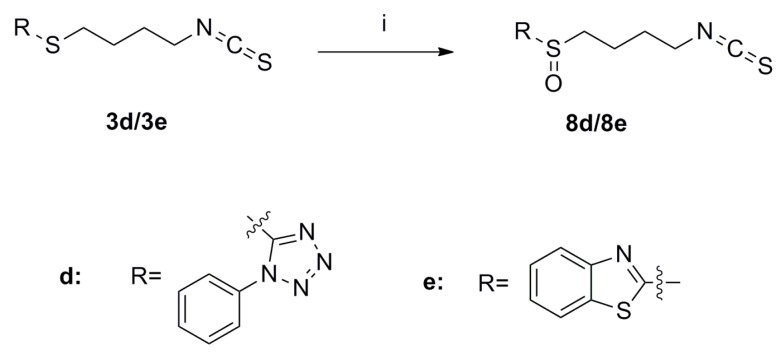
Synthesis of isothiocyanates **8d**–**e**. *Reagents and conditions*: (i) *m*-CPBA, CH_2_Cl_2_, r.t., Yield: **8d**: 20% and **8e**: 40%.

**Scheme 4:**
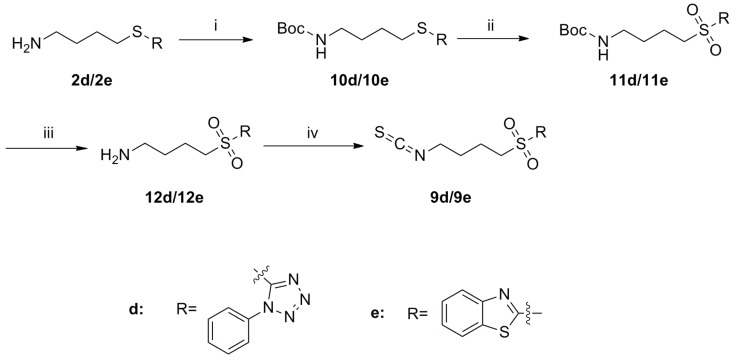
Synthesis of isothiocyanates **9d**–**e**. *Reagents and conditions*: (i) (Boc)_2_O, Et_3_N, CH_2_Cl_2_, r.t., Yield: **10d**: 63% and **10e**: 77%; (ii) TBHP, Ti(O-i-Pr)_4_, CH_2_Cl_2_, r.t., Yield: **11d**: 73% and **11e**: 94%; (iii) CF_3_COOH, CH_2_Cl_2_, NaOH (aq,1 mol/L), r.t., crude; (iv) CSCl_2_, NaOH (aq,1 mol/L), CH_2_Cl_2_, 0 °C to r.t., Yield: **9d**: 15% and **9e**: 22%.

**Scheme 5:**
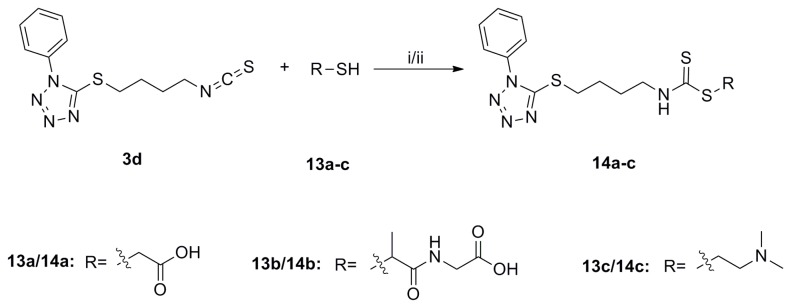
Synthesis of water soluble derivatives **14a**–**c**. *Reagents and conditions*: (i) for **14a**–**b**: DMAP, CH_2_Cl_2_, r.t., Yield: **14a**: 16% and **14b**: 23%; (ii) for **14c**: EtOH, NaOH (aq.), 40 °C, r.t., 20%.

SFN analogues with heterocyclic ring were prepared as shown in Scheme 1, Scheme 2, Scheme 3 and Scheme 4, and water soluble derivatives were prepared as shown in Scheme 5.

As shown in Scheme 1**,** the phthalimide groups in compounds **1a**–**e** were hydrolyzed to yield amines **2a**–**e**, which were then converted to isothiocyanates **3a**–**e** with CSCl_2_ under basic conditions. Sulfides **1a**–**1c** were oxidized by *tert*-butyl hydroperoxide (TBHP) to provide compounds **4a**–**c** and **5a**–**c** (Scheme 2) [20]. These compounds were subsequently hydrolyzed to give amines **6a**–**c** and **7a**–**c**, respectively. Amines **6a**–**c** and **7a**–**c** were further converted to isothiocyanates **8a**–**c** and **9a**–**c**.

Sulfides **3d** and **3e** were oxidized with *m*-chloroperoxybenzoic acid (*m*-CPBA) to provide compounds **8d** and **8e** (Scheme 3) [16]. 

As shown in Scheme 4, protection of the free amines **2d** and **2e** with Boc groups generated compounds **10d** and **10e**, respectively. Sulfides **10d** and **10e** were then oxidized with TBHP to provide sulfones **11d** and **11e**.

Following removal of the Bocgroups in compounds **11d** and **11e**, the free amines **12d** and **12e** were obtained, respectively. Treatment of **12d** and **12e** with CSCl_2_ then converted these compounds to the final isothiocyanate compounds **9d** and **9e**.

However, all of the tetrazole SFN analogues showed low solubility in water, and thus, were further modified to generate water soluble derivatives (Scheme 5). Sulfide **3d** was converted to the water soluble derivatives, **14a**–**c**, via treatment with thiol **13a**–**c** under basic conditions. Unfortunately, water soluble derivatives of sulfoxide **8d** and sulfone **9d** could not be generated, because **8d** and **9d** were unstable under basic conditions, and cleavage of the heterocyclic ring occurred in each case.

### 2.2. Activity of Sulforaphane Analogues and Water Soluble Derivatives

These series of SFN analogues **3a**–**e**, **8a**–**e**, **9a**–**e** and water soluble derivatives **14a**–**c** were evaluated for their activities against MCF-7, SUM-159 and KG-1a cell lines, using SFN and parent molecule **3d** as a positive controls. The results are summarized in Table 1 and Table 2, respectively.

**Figure 1:**
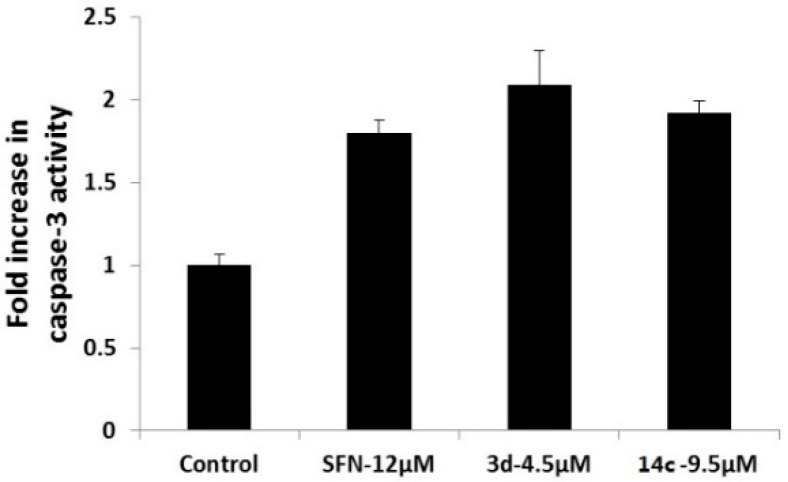
SFN, **3d** and **14c** enhanced caspase-3 activity in SUM-159 cells.

**Figure 2:**
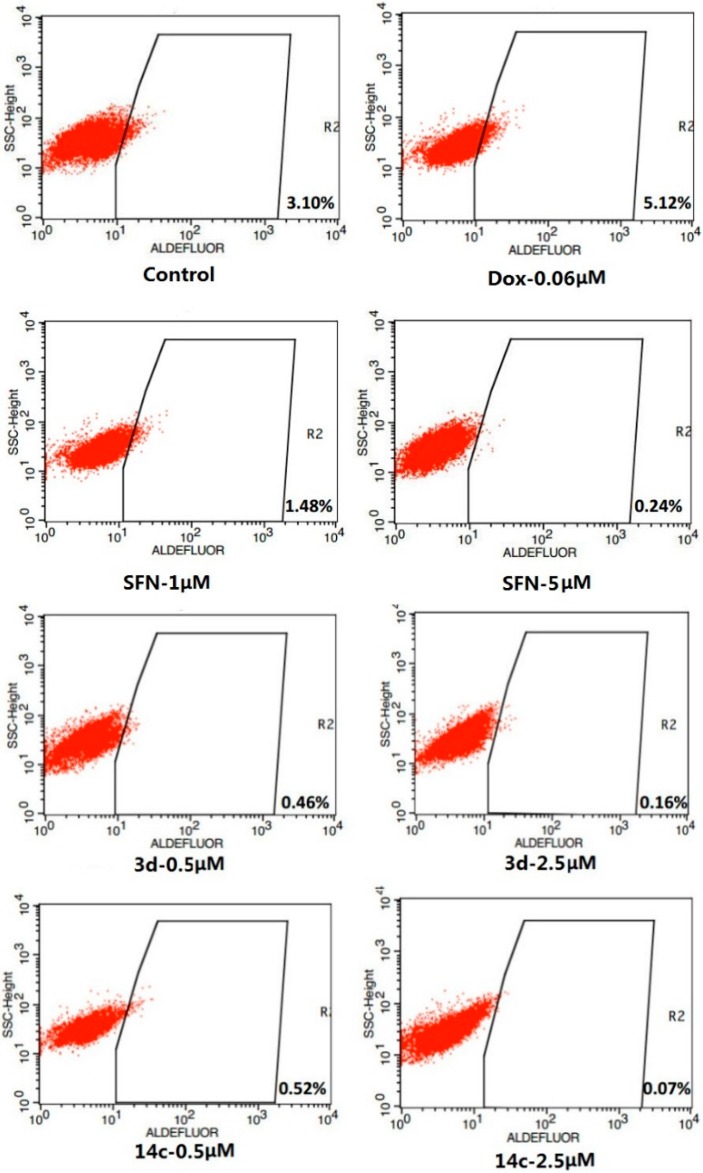
Inhibitory effects of SFN, **3d** and **14c** on ALDH-positive cell subpopulation.

Moreover, the cytotoxicities of the water soluble compounds **14a**–**c** were assayed, and their inhibitory effects on 293T cell line are shown in Table 3. SFN, **3d** and **14c** enhanced caspase-3 activity in SUM-159 cells is shown in Figure 1, and inhibitory effects on ALDH-positive cell subpopulation in SUM-159 cells is shown in Figure 2.

SFN demonstrated a higher potency against the stem cell-like KG-1a cell line (IC_50_ = 8.24 µM) compared with the breast cancer cell lines, MCF-7 (IC_50_ = 24.11 µM) and SUM-159 (IC_50_ = 7.69 µM). The furan compounds, **3a**, **8a** and **9a**, exhibited lower levels of activity compared with SFN against the MCF-7 and SUM-159 cell lines, while sulfoxide **8a** (IC_50_ = 10.87 µM) and sulfone **9a** (IC_50_ = 11.98 µM) exhibited comparable inhibitory activities to SFN against the KG-1a cell line. The methoxypyridine compounds **3b**, **8b** and **9b** exhibited lower activities compared with SFN, yet sulfide **3b** was as potent as SFN against all three of the cell lines evaluated. Among the methoxy-substituted heteroaromatic compounds, **3c**, **8c** and **9c**, sulfide **3c** and sulfoxide **8c** were slightly more potent than SFN against the MCF-7, SUM-159 and KG-1a cell lines, while sulfone **9c** exhibited comparable inhibitory activity compared with SFN. Thetetrazole compounds, **3d**, **8d**, and **9d** exhibited IC_50_ values of 2.66, 4.11, and 1.66 µM, respectively, against the MCF-7 cell line, and were approximately 4.9–13.5 times more potent than SFN (IC_50_ = 24.11 µM). Sulfide **8d** (IC_50_ = 1.54 µM) was approximately 4 times more potent than SFN (IC_50_ = 7.69 µM) against the SUM-159 cell line. Against the KG-1a cell line, **8d** and **9d** exhibited IC_50_ values of 0.51 µM and 0.88 µM, respectively, which were 15.2 and 8.4 times more potent than SFN (IC_50_ = 8.24 µM).All of the thiazole compounds exhibited comparable activity levels to SFN, except sulfide **3e** and sulfone **9e** (IC_50_ > 33.33 µM) were much less active against the SUM-159 cell line than SFN.

Among the five types of heterocyclic SFN analogues that were tested, the tetrazole SFN analogues were the most active against the MCF-7, SUM-159 and KG-1a cell lines. Against the stem cell-like KG-1a cell line, IC_50_ values within 1 µM were observed for **8d** and **9d**.

The activities of water soluble compounds of **3d**, *i.e*., **14a**–**c**, were assayed against the MCF-7, SUM-159 and KG-1a cell lines in an independent experiment, and both SFN and **3d** were applied as the positive controls.The water soluble derivative **14a**, was found to be comparably active against MCF-7 cell lines, and less active against SUM-159 and KG-1a cell lines, and it was less active than the parent molecule **3d** against all three of the cell lines. Compounds **14b** exhibited higher activity than SFN against MCF-7, SUM-159 and KG-1a cell lines, with IC_50_ values of 7.84, 5.96, and 4.73 µM, respectively. Compared with **3d**, compound **14b** exhibited comparable activity against all three cell lines. Compound **14c** exhibited higher activities than SFN against all three cell lines, with IC_50_ values of 7.30, 6.71 and 1.88 µM, respectively. As for inhibitory effect against the stem cell-like KG-1a cell line, water soluble derivative **14c** was about 6.4 times more potent than SFN, and it was also more potent than its parent molecule **3d**.

Compound **14a** (CC_50_ = 10.78 μΜ) exhibited comparable activity to SFN (CC_50_ = 7.84 μΜ) against 293T cell. Compounds **14b** and **14c** exhibited higher activities than SFN against 293T cell, with CC_50_ of 5.39 μΜ and 4.56 μΜ, respectively. Finally, Compounds **14a**–**c** exhibited less effect than the parent molecule **3d** (CC_50_ = 2.12 μΜ) against 293T cell.

In the caspase-3 activity assays, SFN, **3d** and **14c** were found to significantly induce the activation of caspase-3 in the SUM-159 cell line (Figure 1). Based on these results, it appears that SFN, **3d** and **14c** potentially induce apoptosis in cancer cells by increasing caspase-3 activity.

In addition, SFN and doxorubicin (DOX) as well as **3d** and **14c** were assayed for their affects on the ALDH^+^ subpopulation in the SUM-159 cell line (Figure 2). SUM-159 cells were treated with SFN (1 and 5 µM), **3d** (0.5 and 2.5 µM), **14c** (0.5 and 2.5 µM) and DMSO for 4 days and subject to Aldefluor assay and flow cytometry analysis.

Compared to the negative control (containing 3.10% ALDH^+^ cells), treatment with 1 µM SFN significantly decreased the ALDH^+^ population to 1.48%, while 5 µM SFN further reduced the ALDH^+^ population to 0.24% (*p* < 0.05). At 0.5 µM of **3d** or **14c**, the ALDH^+^ population decreased to 0.46% and 0.52%, respectively (*p* < 0.001).At 2.5 µM of **3d** or **14c**, the ALDH^+^ population further decreased to 0.22% and 0.07%, respectively (*p* < 0.001). In contrast, a concentration of 0.06 µM DOX increased the ratio of ALDH^+^ cells to 5.12%.

In general, conventional drugs such as DOX are usually less active against ALDH^+^ cells, thereby leading to an increase in this cell population. In contrast, SFN, **3d**, and **14c** were found to selectively inhibit the growth of ALDH^+^ cells at concentrations that were 10-fold lower than their IC_50_ values against the SUM-159 cell.

## 3.Experimental Section

### 3.1.General Information

^1^H- and ^13^C-NMR spectra were obtained using a AV 400 spectrometer (Bruker, Madison, WI, USA) using CDCl_3_ as the solvent.Chemical shifts are reported in parts per million (ppm) relative to either a tetramethylsilane internal standard or solvent signals. Data are reported as follows: chemical shift, multiplicity (s = singlet, d = doublet, t = triplet, q = quartet, br. = broad, m = multiplet), coupling constants and integration. 

### 3.2. General Procedure for the Synthesis of Compounds ***1a**–**e***

Compounds **1a**–**e** were prepared according to a literature methid according to the literature method [19].

*2-(4-((Furan-2-ylmethyl)thio)bu*t*yl)isoindoline-1,3-dione* (**1a**): yield 90%, white solid; ^1^H-NMR (CDCl_3_) δ 7.86 (q, *J*_1_ = 3.2 Hz, *J*_2_ = 5.6 Hz, 2H), 7.73 (q, *J*_1_ = 2.8 Hz, *J*_2_ = 5.2 Hz, 2H), 7.34 (d, *J* = 0.8 Hz, 1H), 6.29 (t, *J* = 3.2 Hz, 1H), 6.18 (d, *J* = 2.8 Hz, 1H), 3.69–3.72 (m, 4H), 2.56 (t, *J* = 7.2 Hz, 2H), 1.75–1.83 (m, 2H), 1.60–1.66 (m, 3H); MS: [M + Na]^+^ 338.14.

*2-(4-((5-Methoxy-3H-imidazo*[4,5-b]*pyridin-2-yl)thio)butyl)isoindoline-1,3-dione* (**1b**): yield 89%, yellow solid; ^1^H-NMR (CDCl_3_) δ 7.86 (q, *J*_1_ = 3.2 Hz, *J*_2_ = 5.6 Hz, 2H), 7.72–7.75 (m, 3H), 6.64 (d, *J* = 8.8 Hz, 1H), 3.98 (s, 3H), 3.75 (t, *J* = 6.8 Hz, 2H), 3.39 (t, *J* = 6.8 Hz, 2H), 1.81–1.93 (m, 4H); MS: [M + H]^+^ 383.71.

*2-(4-((6-Methoxy-1H-benzo[d]imidazol-2-yl)thio)butyl)isoindoline-1,3-dione* (**1c**): yield 74.1%, yellow solid; ^1^H-NMR (CDCl_3_) δ 7.84 (q, *J*_1_ = 3.6 Hz, *J*_2_ = 5.6 Hz, 2H), 7.72 (q, *J*_1_ = 3.2 Hz, *J*_2_ = 5.2 Hz, 2H), 7.40 (d, *J* = 8.8 Hz, 1H), 7.02 (s, 1H), 6.84 (dd, *J*_1_ =2.0 Hz, *J*_2_ = 8.8 Hz, 1H), 3.84 (s, 3H), 3.72 (t, *J* = 6.4 Hz, 2H), 3.33 (t, *J* = 6.8 Hz, 2H), 1.79–1.90 (m, 4H); ^13^C-NMR (CDCl_3_) δ 168.59, 156.28, 149.16, 134.09, 132.07, 123.34, 111.55, 55.94, 37.37, 32.36, 27.61, 27.01; MS: [M + H]^+^ 382.19; Mp: 87.6–88.9 °C.

*2-(4-((5-Phenyl-1H-tetrazol-1-yl)thio)butyl)isoindoline-1,3-dione* (**1d**): yield 87%, yellow solid; ^1^H-NMR (CDCl_3_) δ 7.86 (q, *J*_1_ = 3.2 Hz, *J*_2_ = 5.6 Hz, 2H), 7.74 (q, *J*_1_ = 3.2 Hz, *J*_2_ = 5.6 Hz, 2H), 7.54–7.60 (m, 5H), 3.76 (t, *J* = 6.8 Hz, 2H), 3.47 (t, *J* = 6.8 Hz, 2H), 1.84–1.98 (m, 4H).

*2-(4-(Benzo[d]thiazol-2-ylthio)butyl)isoindoline-1,3-dione* (**1e**): yield 92%, yellow solid; ^1^H-NMR (CDCl_3_) δ 7.85–7.91 (m, 3H), 7.72–7.77 (m, 3H), 7.44 (t, *J* = 8.0 Hz, 1H), 7.32 (t, *J* = 8.0 Hz, 1H), 3.78 (t, *J* = 6.4 Hz, 2H), 3.43–3.47 (m, 2H), 1.91–1.94 (m, 4H); ^13^C-NMR (CDCl_3_) δ 168.50, 166.81, 153.40, 135.34, 134.07, 132.21, 126.11, 124.28, 123.37, 121.65, 121.05, 37.48, 33.01, 27.79, 26.75; MS: [M + H]^+^ 369.12; Mp: 87.5–88.6 °C.

### 3.3. General Procedure for the Synthesis of Amines ***2a**–**e***, ***6a**–**c*** and ***7a**–**c***

To a solution of compound **1** (1 mmol) in anhydrous MeOH (10 mL) was added methylamine (10 mL, 40% water solution) at room temperature. The mixture was stirred at room temperature overnight, and the solvent was removed under reduced pressure to give crude residue, which was purified by columnchromatography to obtain **2**.The synthesis of **6** and **7** were similar to that of **2**.

*4-((Furan-2-ylmethyl)thio)butan-1-amine* (**2a**): yield 71%, yellow oil; ^1^H-NMR (CDCl_3_) δ 7.35 (dd, *J*_1_ = 0.8 Hz, *J*_2_ = 2.0 Hz, 1H), 6.30 (dd, *J*_1_ = 2.0 Hz, *J*_2_ = 3.2 Hz, 1H), 6.16 (d, *J* = 3.2 Hz, 1H), 3.71 (s, 2H), 2.68 (t, *J* = 6.8 Hz, 2H), 2.51 (t, *J* = 7.2 Hz, 2H), 1.64–1.47 (m, 4H).

*4-((5-Methoxy-3H-imidazo*[4,5-b]*pyridin-2-yl)thio)butan-1-amine* (**2b**): yield 71%, white oil; ^1^H-NMR (CDCl_3_) δ 7.69 (d, *J* = 8.6 Hz, 1H), 6.59 (d, *J* = 8.6 Hz, 1H), 3.95 (s, 3H), 3.25 (t, *J* = 7.2 Hz, 2H), 2.85 (t, *J* = 6.7 Hz, 2H), 1.89 (td, *J*_1_ = 6.5 Hz, *J*_2_ = 8.0 Hz, *J*_3_ = 14.8 Hz, 2H), 1.69–1.62 (m, 2H); MS: [M + H]^+^ 253.24, [M − H]^−^ 251.25.

*4-((6-Methoxy-1H-benzo[d]imidazol-2-yl)thio)butan-1-amine* (**2c**): yield 71%, white solid; ^1^H-NMR (MeOH) δ 7.30 (d, *J* = 8.7 Hz, 1H), 6.93 (d, *J* = 2.4 Hz, 1H), 6.77 (dd, *J*_1_ = 2.4 Hz, *J*_2_ = 8.8 Hz, 1H), 3.76 (s, 3H), 3.17 (t, *J* = 7.2 Hz, 2H), 2.60 (t, *J* = 7.1 Hz, 2H), 1.78–1.70 (d, *J* = 7.2 Hz, 2H), 1.63–1.49 (m, 2H); ^13^C-NMR (CDCl_3_) δ 156.05, 149.93, 140.08, 134.69, 114.74, 111.06, 97.54, 55.95, 41.14, 32.09, 31.39, 27.11; MS: [M + H]^+^ 252.20; Mp: 105.4–106.9°C.

*4-((5-Phenyl-1H-tetrazol-1-yl)thio)butan-1-amine* (**2d**): yield 70%, yellow oil; ^1^H-NMR (CDCl_3_) δ 7.59–7.52 (m, 5H), 3.41 (t, *J* = 7.4 Hz, 2H), 2.75 (t, *J* = 7.0 Hz, 2H), 1.91–1.86 (m, 2H), 1.63–1.58 (m, 2H); MS: [M + H]^+^ 250.15.

*4-(Benzo[d]thiazol-2-ylthio)butan-1-amine*(**2e**): yield 85%, yellow oil; ^1^H-NMR (CDCl_3_) δ 7.59 (d, *J* = 8.1 Hz, 1H), 7.48 (d, *J* = 7.9 Hz, 1H), 7.14 (t, *J* = 7.7 Hz, 1H), 7.05–6.98 (m, 1H), 3.10 (t, *J* = 7.3 Hz, 2H), 1.65–1.58 (m, 2H), 1.41–1.33 (m, 2H), 1.19 (bs, 2H); ^13^C-NMR (CDCl_3_) δ 167.16, 153.41, 135.28, 126.11, 124.26, 121.56, 121.03, 41.67, 33.40, 32.77, 26.80; MS: [M + H]^+^ 239.26.

*4-((Furan-2-ylmethyl)sulfinyl)butan-1-amine* (**6a**): yield 48%, yellow oil; ^1^H-NMR (CDCl_3_): δ 7.41 (s, 1H), 6.39 (s, 2H), 4.04 (d, *J* = 3.0 Hz, 2H), 2.72 (t, *J* = 6.9 Hz, 2H), 2.68 (t, *J* = 7.6 Hz, 2H), 1.75–1.87 (m, 2H), 1.51–1.62 (m, 4H); ^13^C-NMR (CDCl_3_) δ 143.96, 143.42, 111.20, 51.25, 50.54, 41.42, 32.39, 19.79; MS: [M + H]^+^ 202.08.

*4-((5-Methoxy-3H-imidazo*[4,5-b]*pyridin-2-yl)sulfinyl)butan-1-amine* (**6b**): yield 47%, yellow oil; ^1^H-NMR (CD_3_OD) δ 7.78 (d, *J =* 8.6 Hz, 1H), 6.52 (d, *J =* 8.6 Hz, 1H), 5.44 (s, 1H), 3.88 (s, 3H), 3.28–3.23 (m, 2H), 2.88–2.75 (m, 2H), 1.78–1.61 (m, 4H); ^13^C-NMR (CD_3_OD) δ 162.12, 159.16, 155.91, 133.19, 129.19, 106.07, 53.82, 53.29, 40.52, 28.81, 20.70; MS: [M + H]^+^ 269.25.

*4-((6-Methoxy-1H-benzo[d]imidazol-2-yl)sulfinyl)butan-1-amine* (**6c**): yield 77%, white oil; ^1^H-NMR (CD_3_OD) δ 7.36 (d, *J =* 8.9 Hz, 1H), 6.96 (d, *J =* 2.2 Hz, 1H), 6.73 (dd, *J =* 8.9, 2.3 Hz, 1H), 3.67 (s, 3H), 3.21 (s, 1H), 3.19–3.12 (m, 2H), 2.66–2.60 (m, 2H), 1.69–1.47 (m, 4H); ^13^C-NMR (CD_3_OD) δ 158.23, 155.34, 142.58, 138.03, 118.61, 114.48, 98.57, 56.12, 54.11, 40.90, 29.89, 20.50; MS: [M + H]^+^ 268.23.

*4-((Furan-2-ylmethyl)sulfonyl)butan-1-amine* (**7a**): yield 64%, white solid; ^1^H-NMR (CDCl_3_): δ 7.45 (d, *J =* 1.6 Hz, 1H), 6.51 (d, *J =* 3.2 Hz, 1H), 6.43 (dd, *J*_1_ = 2.0 Hz, *J*_2_ = 3.2 Hz, 1H), 4.29 (s, 2H), 2.93 (t, *J* = 8.0 Hz, 2H), 2.71 (t, *J* = 7.2 Hz, 2H), 1.82–1.90 (m, 2H), 1.52–1.59 (m, 2H); ^13^C-NMR (CDCl_3_) δ 143.91, 142.64, 112.33, 111.63, 52.70, 51.85, 41.49, 32.31, 19.45; MS: [M + H]^+^ 218.15; Mp: 105.9–107.1 °C.

*4-((5-Methoxy-3H-imidazo*[4,5-b]*pyridin-2-yl)sulfonyl)butan-1-amine* (**7b**): yield 46%, yellow oil; ^1^H-NMR (CD_3_OD) δ 7.85 (d, *J =* 8.7 Hz, 1H), 6.61 (d, *J =* 8.7 Hz, 1H), 3.93 (s, 3H), 3.39 (t, *J =* 6.9 Hz, 2H), 3.35 (s, 1H), 2.92 (t, *J =* 6.8 Hz, 2H), 2.68 (d, *J =* 2.0 Hz, 1H), 2.55 (s, 1H), 1.86–1.71 (m, 4H); ^13^C-NMR (CD_3_OD) δ 162.79, 155.12, 155.07, 132.83, 130.14, 107.59, 55.02, 53.75, 40.10, 27.18, 21.07; MS: [M + H]^+^ 285.23.

*4-((6-Methoxy-1H-benzo[d]imidazol-2-yl)sulfonyl)butan-1-amine* (**7c**): yield 60%, white solid; ^1^H-NMR (DMSO-*d*_6_) δ 7.40 (d, *J =* 8.7 Hz, 1H), 6.99 (d, *J =* 2.3 Hz, 1H), 6.69 (dd, *J =* 8.8, 2.4 Hz, 1H), 3.73 (s, 3H), 3.41–3.33 (m, 2H), 2.79 (t, *J =* 7.1 Hz, 2H), 1.74–1.69 (m, 2H), 1.64–1.59 (m, 2H); ^13^C-NMR (DMSO-*d*_6_) δ 154.95, 154.81, 144.21, 139.14, 118.27, 111.25, 98.92, 55.23, 52.74, 38.70, 26.66, 19.55; MS: [M + H]^+^ 284.31; Mp: 138.6–139.9 °C.

### 3.4. General Procedure for the Synthesis of Sulfides ***3a**–**e***, Sulfoxides ***8a**–**c*** and Sulfones ***9a**–**c***

To a solution of **2** (1 mmol) and NaOH (1 mol/L, 1.66 mmol) in anhydrous CH_2_Cl_2_ (10 mL) was added CSCl_2_ (2.55 mmol) at 0 °C. The mixture was stirred for 20 min, warmed to room temperature, and continued to stir for 3 h. The reaction mixture was diluted with brine (10 mL) and CH_2_Cl_2_ (10 mL), and extracted with CH_2_Cl_2_(10 mL × 2). The organic layer was dried over anhydrous Na_2_SO_4_, and solvent was removed under reduced pressure. The residue was purified by columnchromatography to obtain isothiocyanate **3**. The synthesis of compounds **8** and **9** were similar to **3**.

*2-(((4-Isothiocyanatobutyl)thio)methyl)furan* (**3a**): yield 78%, yellow oil; ^1^H-NMR (CDCl_3_): δ 7.36 (d, *J*_1_ = 1.8 Hz, *J*_2_ = 0.9 Hz,1H), 6.31 (dd, *J*_1_ = 3.2 Hz, *J*_2_ = 1.9 Hz, 1H), 6.18 (d, *J* = 2.8 HZ, 1H), 3.72 (s, 2H), 3.51 (t, *J* = 6.4 Hz, 2H), 2.53 (t, *J* = 7.0 Hz, 2H), 1.81–1.74 (m, 2H), 1.71–1.64 (m, 2H); ^13^C-NMR (CDCl_3_): δ 151.5, 142.2, 130.2, 110.5, 107.6, 44.7, 30.8, 28.9, 28.3, 26.0; MS:[M + H]^+^ 228.2.

*2-((4-Isothiocyanatobutyl)thio)-5-methoxy-3H-imidazo*[4,5-b]*pyridine* (**3b**): yield 66%, yellow soild; ^1^H-NMR (CDCl_3_) δ 9.75 (bs,1H), 7.76 (d, *J* = 8.8 Hz, 1H), 6.63 (d, *J* = 8.4 Hz, 1H), 3.96 (s, 3H), 3.51 (t, *J* = 6.4 Hz, 2H), 3.34 (t, *J* = 6.4 Hz, 2H), 1.93–1.87 (m, 2H), 1.85–1.77 (m, 2H); ^13^C-NMR (CDCl_3_): δ 161.3, 149.2, 130.5, 127.6, 126.0, 105.6, 54.2, 44.7, 31.7, 28.9, 26.8; MS: [M + H]^+^ 295.14. Mp: 79.3–81.6 °C.

*2-((4-Isothiocyanatobutyl)thio)-6-methoxy-1H-benzo[d]imidazole* (**3c**): yield 37%, yellow soild; ^1^H-NMR (CDCl_3_) δ 7.40 (d, *J* = 8.8 Hz, 1H), 7.01 (s, 1H), 6.85 (dd, *J*_1_ = 8.8 Hz, *J*_2_ = 2.4 Hz, 1H), 3.83 (s, 3H), 3.53 (t, *J* = 6.4 Hz, 2H), 3.32 (t, *J* = 6.8 Hz, 2H), 1.94–1.79 (m, 4H); ^13^C-NMR (CDCl_3_): δ 156.5, 148.8, 111.7, 56.0, 44.7, 32.2, 28.9, 26.9. HRMS-ESI (+) for C_13_H_16_N_3_OS_2_, calculated 294.0735, found 294.0745 [M + H]^+^; Mp: 58.9–60.4°C.

*1-((4-Isothiocyanatobutyl)thio)-5-phenyl-1H-tetrazole* (**3d**): yield 81%, yellow solid; ^1^H-NMR (CDCl_3_): δ 7.52–7.58 (m, 5H), 3.59 (t, *J* = 6.4 Hz, 2H), 3.43 (t, *J* = 7.0 Hz, 2H), 1.96–2.03 (m, 2H), 1.83–1.90 (m, 2H); ^13^C-NMR (CDCl_3_): δ 154.0, 133.7, 130.2, 129.9, 123.9, 44.5, 32.3, 28.9, 26.4; HRMS-ESI (+) for C_12_H_14_N_5_S_2_, calculated 292.0691, found 292.0964 [M + H]^+^; Mp: 52.8–55.0 °C.

*2-((4-Isothiocyanatobutyl)thio)benzo[d]thiazole* (**3e**): yield 64%, yellow soild; ^1^H-NMR (CDCl_3_) δ 7.87 (d, *J* = 8.4 Hz, 1H), 7.75 (d, *J* = 8.0 Hz, 1H), 7.41 (t, *J* = 7.6 Hz, 1H), 7.29 (t, *J* = 7.6 Hz, 1H), 3.57 (t, *J* = 6.4 Hz, 2H), 3.38 (t, *J* = 7.2 Hz, 2H), 1.84–2.00 (m, 4H); ^13^C-NMR (CDCl_3_) δ 166.3, 153.2, 135.3, 126.1, 124.4, 121.6, 121.1, 44.6, 32.5, 28.9, 26.5; HRMS-ESI (+) for C_12_H_13_N_2_S_3_, ca lculated 281.0241, found 281.0250 [M + H]^+^; Mp: 60.2–61.9 °C.

*2-(((4-Isothiocyanatobutyl)sulfinyl)methyl)furan* (**8a**): yield 53%, yellow oil; ^1^H-NMR (CDCl_3_): δ 7.44 (s, 1H), 6.41 (s, 2H), 4.08 (d, *J* = 7.6 Hz, 2H), 3.57 (t, *J* = 6.0 Hz, 2H), 2.71–2.60 (m, 2H), 1.95–1.78 (m, 4H); ^13^C-NMR (CDCl_3_): δ 143.6, 111.4, 111.3, 50.8, 50.5, 44.6, 29.1, 19.9; HRMS-ESI (+) for C_10_H_13_NO_2_S_2_Na, calculated 266.0285, found 266.0294 [M + Na]^+^.

*2-((4-Isothiocyanatobutyl)sulfinyl)-5-methoxy-3H-imidazo*[4,5-b]*pyridine* (**8b**): yield 58%, yellow solid; ^1^H-NMR (CDCl_3_): δ 7.89 (d, *J* = 8.8 Hz, 1H),6.78 (d, *J* = 8.8 Hz, 1H), 4.00 (s, 3H), 3.56 (t, *J* = 5.6 Hz, 2H), 3.37–3.43 (m, 2H), 2.18–2.04 (m, 1H), 1.94–1.78 (m, 3H); ^13^C-NMR (CDCl_3_): δ 162.6, 150.8, 131.5, 127.8, 108.9, 54.1, 53.5, 44.7, 29.0, 19.1; HRMS-ESI (+) for C_12_H_14_N_4_O_2_S_2_Na, calculated 333.0456, found 333.0467 [M + Na]^+^; Mp: 144.5–146.1 °C.

*2-((4-Isothiocyanatobutyl)sulfinyl)-6-methoxy-1H-benzo[d]imidazole* (**8c**): yield 51%, yellow soild; ^1^H-NMR (CDCl_3_): δ 7.58 (d, *J* = 8.9 Hz, 1H), 7.09 (d, *J* = 2.4 Hz, 1H), 7.00 (dd, *J*_1_ = 8.8 Hz, *J*_2_ = 2.0 Hz, 1H), 3.87 (s, 3H), 3.56–3.52 (m, 2H), 3.44–3.28 (m, 2H), 2.10–2.02 (m, 1H), 1.92–1.76 (m, 3H); ^13^C-NMR (CDCl_3_): δ 157.6, 151.0, 131.5, 114.5, 56.0, 53.8, 44.7, 29.0, 19.3; HRMS-ESI (+) for C_13_H_15_N_3_O_2_S_2_Na, calculated 332.0504, found 332.0488 [M + Na]^+^; Mp: 93.4–95.2 °C.

*2-(((4-Isothiocyanatobutyl)sulfonyl)methyl)furan* (**9a**): yield 63%, white solid; ^1^H-NMR (CDCl_3_): δ 7.49 (s, 1H) 6.54 (d, *J* = 3.2 Hz, 1H), 6.48–6.44 (m, 1H), 4.33 (s, 2H), 3.57 (t, *J* = 6.4 Hz, 2H), 2.96 (t, *J* = 7.6 Hz, 2H), 2.00–1.93 (m, 2H), 1.88–1.82 (m, 2H); ^13^C-NMR (CDCl_3_): δ 144.1, 142.4, 112.6, 111.8, 53.1, 51.1, 44.6, 28.8, 19.5; HRMS-ESI (+) for C_10_H_13_NO_3_S_2_Na, calculated 282.0235, found 282.0242 [M + Na]^+^; Mp: 67.7–68.9 °C.

*2-((4-Isothiocyanatobutyl)sulfonyl)-5-methoxy-3H-imidazo*[4,5-b]*pyridine* (**9b**): yield 80%, white soild; ^1^H-NMR (CDCl_3_): δ 8.00 (d, *J* = 8.8 Hz, 1H), 6.85 (d, *J* = 8.8 Hz, 1H), 3.98 (s, 3H) , 3.54–3.58 (m, 4H), 2.03–1.95 (m, 2H), 1.89–1.82 (m, 2H); ^13^C-NMR (CDCl_3_): δ 171.6, 163.9, 145.1, 131.3, 110.8, 54.3, 54.1, 44.5, 28.5, 19.9; HRMS-ESI (+) for C_12_H_14_N_4_O_3_S_2_Na, calculated 349.0405, found 349.0414 [M + Na]^+^; Mp: 65.5–67.3 °C.

*2-((4-Isothiocyanatobutyl)sulfonyl)-6-methoxy-1H-benzo[d]imidazole* (**9c**): yield 56%, yellow soild; ^1^H-NMR (CDCl_3_) δ 7.75–7.56 (m, 1H), 7.06–7.08 (m, 2H), 3.86 (s, 3H), 3.52–3.57 (m, 4H), 1.95–2.03 (m, 2H), 1.82–1.89 (m, 2H); ^13^C-NMR (CDCl_3_): δ 158.9, 14598, 131.4, 116.7, 56.0, 54.2, 44.6, 28.5, 20.0; HRMS-ESI (+) for C_13_H_15_N_3_O_3_S_2_Na, calculated 348.0453, found 348.0467 [M + Na]^+^; Mp: 112.2–113.5 °C.

### 3.5. General Procedure for the Synthesis of Amides ***4a**–**c*** and ***5a**–**c***

To a solution of compound **1** (1 mmol) in anhydrous CH_2_Cl_2_ (20 mL) under an argon atmosphere was added Ti(O-*i*-Pr)_4_ (1 mmol), and the reaction mixture was stirred at room temperature for 15 min. Then the reaction mixture was cooled to−20 °C for 20 min, and a solution of TBHP (2 mmol) in anhydrous CH_2_Cl_2_ (3.2 mL) was added slowly. The mixture was stirred at −20 °C for 8–12 h, and water (20 mL) was added, the reaction mixture was stirred for 1 h, and the resulting gel was dissolved with ethyl acetate (30 mL× 2). After remove the solvent under reduced pressure, and obtain crude **4** or **5**, which was purified withcolumnchromatography.

*2-(4-((Furan-2-ylmethyl)sulfinyl)butyl)isoindoline-1,3-dione* (**4a**): yield 90%, white soild; ^1^H-NMR (CDCl_3_): δ 7.84 (dd, *J*_1_ = 5.6 Hz, *J*_2_ = 3.2 Hz, 2H), 7.72 (dd, *J*_1_ = 5.5 Hz, *J*_2_ = 3.0 Hz, 2H), 7.41 (dd, *J*_1_ = 0.8 Hz, *J*_2_ = 2.0 Hz, 1H), 6.40–6.37 (m, 2H), 4.05 (d, *J* = 2.8 Hz, 2H), 3.71–3.74 (m, 2H), 2.62–2.74 (m, 2H), 1.76–1.89 (m, 4H); ^13^C-NMR (CDCl_3_) δ 168.31, 143.93, 143.48, 134.05, 132.04, 123.30, 111.26, 50.96, 50.69, 37.20, 27.82, 19.81; MS: [M + H]^+^ 332.23; Mp: 75.6–77.1°C.

*2-(4-((5-Methoxy-3H-imidazo*[4,5-b]*pyridin-2-yl)sulfinyl)butyl)isoindoline-1,3-dione* (**4b**): yield 58%, white soild; ^1^H-NMR (CDCl_3_) δ 7.86 (d, *J* = 8.8 Hz, 1H), 7.79 (dd, *J*_1_ = 2.0 Hz, *J*_2_=3.6 Hz, 2H), 7.70 (q, *J*_1_ = 2.0 Hz, *J*_2_ = 3.6 Hz, 2H), 6.77 (d, *J* = 6.0 Hz, 1H), 4.00 (s, 3H), 3.70 (t, *J* = 4.4 Hz, 2H), 3.39–3.44 (m, 1H), 3.28–3.33 (m, 1H), 2.00–2.08 (m, 2H), 1.85–1.93 (m, 2H); ^13^C-NMR (CDCl_3_) δ 168.34, 162.54, 134.08, 131.96, 130.97, 123.32, 109.13, 108.32, 54.04, 37.17, 29.81, 27.60, 19.34; MS: [M + H]^+^ 399.18; Mp: 80.6–81.7 °C.

*2-(4-((6-Methoxy-1H-benzo[d]imidazol-2-yl)sulfinyl)butyl)isoindoline-1,3-dione* (**4c**): yield 82%, white soild; ^1^H-NMR (CDCl_3_) δ 11.84 (s, 1H), 7.65–7.62 (m, 2H), 7.57–7.55 (m, 2H), 7.43 (bs, 1H), 6.83 (d, *J* = 8.0 Hz, 2H), 3.75 (s, 3H), 3.57 (t, *J =* 6.9 Hz, 2H), 3.37–3.14 (m, 2H), 1.92–1.63 (s, 4H); ^13^C-NMR (CDCl_3_) δ 168.35, 157.42, 151.15, 134.07, 131.96, 123.33, 114.39, 55.90, 54.19, 37.16, 27.58, 19.37; MS: [M + H]^+^ 398.40; Mp: 141.3–142.8°C.

*2-(4-((Furan-2-ylmethyl)sulfonyl)butyl)isoindoline-1,3-dione* (**5a**): yield 60%, white soild; ^1^H-NMR (CDCl_3_) δ 7.84 (dd, *J =* 5.4, 3.0 Hz, 2H), 7.72 (dd, *J =* 5.4, 3.1 Hz, 2H), 7.42 (s, 1H), 6.51 (d, *J =* 3.2 Hz, 1H), 6.41–6.40 (m, 1H), 4.30 (s, 2H), 3.71 (t, *J =* 6.2 Hz, 2H), 2.98 (t, *J =* 7.2 Hz, 2H), 1.89–1.80 (m, 4H); ^13^C-NMR (CDCl_3_) δ 168.42, 143.98, 142.56, 134.20, 132.12, 123.46, 112.44, 111.67, 52.88, 51.52, 37.06, 27.49, 19.39; MS: [M + H]^+^ 348.22; Mp: 81.9–83.2°C.

*2-(4-((5-Methoxy-3H-imidazo*[4,5-b]*pyridin-2-yl)sulfonyl)butyl)isoindoline-1,3-dione* (**5b**): yield 95%, white soild; ^1^H-NMR (CDCl_3_): δ 7.94 (d, *J* = 8.8 Hz, 1H), 7.76 (q, *J*_1_ = 5.2 Hz, *J*_2_ = 3.2 Hz, 2H), 7.68 (dd, *J*_1_ = 3.2 Hz, *J*_2_ = 5.6 Hz, 2H), 6.82 (d, *J* = 9.2 Hz, 1H), 3.99 (s, 3H), 3.68 (t, *J* = 6.4 Hz, 2H), 3.56 (t, *J* = 6.8 Hz, 2H), 1.80–1.97 (m, 4H); ^13^C-NMR (DMSO-*d*_6_) δ 167.90, 162.32, 146.48, 134.35, 131.49, 122.97, 109.50, 53.45, 48.65, 36.64, 26.45, 19.66; MS: [M + H]^+^ 415.17; Mp: 91.3–92.8 °C.

*2-(4-((6-Methoxy-1H-benzo[d]imidazol-2-yl)sulfonyl)butyl)isoindoline-1,3-dione* (**5c**): yield 60%, white soild; ^1^H-NMR (CDCl_3_): δ 7.69–7.72 (m, 2H), 7.64–7.67 (m, 2H), 7.59 (d, *J* = 8.8 Hz, 1H), 7.05 (s, 1H), 7.00 (dd, *J*_1_ = 9.0 Hz, *J*_2_ = 2.4 Hz, 1H), 3.82 (s, 3H), 3.66 (t, *J* = 6.6 Hz, 2H), 3.56 (t, *J* = 6.8 Hz, 2H), 1.72–1.92 (m, 4H); ^13^C-NMR (CDCl_3_) δ 168.42, 164.11, 144.77, 144.14, 134.18, 132.52, 132.01, 130.38, 123.44, 110.29, 54.42, 54.32, 36.88, 27.10, 19.80; MS: [M + H]^+^ 414.18; Mp: 101.5–102.4°C.

### 3.6. General Procedure for the Synthesis of Sulfoxide Compounds ***8d*** and ***8e***

To a solution of compound **3** (1 mmol) in anhydrous CH_2_Cl_2_ (20 mL) under an argon atmosphere was added MCPBA (2 mmol) at 0 °C, and the resulting reaction mixture was stirred at room temperature for overnight. Saturated sodium bicarbonate solution was added, and the aqueous layer was extracted with CH_2_Cl_2_ (10 mL × 2). The combined organic layer was dried over anhydrous Na_2_SO_4_, and the solvent was removed under reduced pressure, the residue was purified with columnchromatography to obtain **8**.

*1-((4-Isothiocyanatobutyl)sulfinyl)-5-phenyl-1H-tetrazole* (**8d**): yield 20%, yellow soild; ^1^H-NMR (CDCl_3_): δ 7.73–7.70 (m, 2H), 7.65–7.60 (m, 3H), 3.72 (dt, *J*_1_ = 13.4 Hz, *J*_2_ = 7.4 Hz, 1H), 3.61 (t, *J* = 6.3 Hz, 2H), 3.51–3.58 (m, 1H), 1.99–2.07 (m, 2H), 1.89–1.96 (m, 2H); ^13^C-NMR (CDCl_3_) δ 160.0, 133.1, 131.5, 130.2, 125.1, 51.8, 44.6, 29.0, 19.9; HRMS-ESI (+) for C_12_H_14_N_5_OS_2_, calculated 308.0640, found 308.0644 [M + H]^+^; Mp: 69.5–71.2 °C.

*2-((4-Isothiocyanatobutyl)sulfinyl)benzo[d]thiazole* (**8e**): yield 40%, yellow soild; ^1^H-NMR (CDCl_3_): δ 8.08 (d, *J* = 8.0 Hz, 1H), 8.01 (d, *J* = 8.0 Hz, 1H), 7.58 (m, 1H), 7.50 (m, 1H), 3.57 (t, *J* = 6.4 Hz, 2H), 3.36–3.20 (m, 2H), 2.15–2.06 (m, 1H), 1.81–1.96 (m, 3H); ^13^C-NMR (CDCl_3_) δ 176.9, 153.9, 136.0, 131.3, 127.1, 126.3, 124.0, 122.4, 55.2, 44.5, 28.9, 19.1; HRMS-ESI (+) for C_12_H_12_N_2_OS_3_Na, calculated 319.000, found 319.0014 [M + Na]^+^; Mp: 79.5–81.1 °C.

### 3.7. General Procedure for the Synthesis of Tert-Butyl Carbonate Sulfide Compounds ***10d*** and ***10e***

To a solution of compound **2** (1 mmol) in anhydrous CH_2_Cl_2_ (20 mL) was added Et_3_N (2 mmol), and the reaction mixture was stirred at room temperature for 30 min. (Boc)_2_O was added, and then the mixture was stirred at room temperature for another 8 h. Water (10 mL) was added to quench the reaction, and the aqueous layer was extracted with CH_2_Cl_2_ (10 mL × 2). The combined organic layer was dried over anhydrous Na_2_SO_4_, and the solvent was removed under reduced pressure, and the crude residue was purified with columnchromatography to obtain **10**.

*tert-Butyl (4-((5-phenyl-1H-tetrazol-1-yl)thio)butyl)carbamate* (**10d**): yield 63%, yellow oil; ^1^H-NMR (CDCl_3_) δ 7.59–7.53 (m, 5H), 3.40 (t, *J* = 7.6 Hz, 2H), 3.17 (d, *J* = 6.2 Hz, 2H), 1.92–1.84 (m, 2H), 1.61–1.68 (m, 2H), 1.43 (s, 9H); ^13^C-NMR (CDCl_3_) δ 156.04, 154.35, 133.74, 130.18, 129.86, 123.89, 79.25, 39.86, 32.86, 29.13, 28.47, 26.53; MS: [M + H]^+^ 350.20.

*tert-Butyl (4-(benzo[d]thiazol-2-ylthio)butyl)carbamate* (**10e**): yield 77%, yellow oil; ^1^H-NMR (CDCl_3_) δ 7.89 (d, *J* = 8.0 Hz, 1H), 7.75 (d, *J* = 8.0 Hz, 1H), 7.41 (m, 1H), 7.35–7.27 (m, 1H), 3.36 (t, *J* = 7.2 Hz, 2H), 3.21 (m, 2H), 1.85–1.92 (m, 2H), 1.64–1.71 (m, 2H), 1.44 (s, 9H); ^13^C-NMR (CD_3_OD) δ 169.29, 158.51, 154.37, 136.17, 127.34, 125.54, 122.26, 122.11, 79.88, 40.66, 34.03, 30.00, 28.77, 27.79; MS: [M + H]^+^ 339.16.

### 3.8. General Procedure for the Synthesis of Tert-Butyl Carbonate Sulfone Compounds ***11d*** and ***11e***

To a solution of compound **10** (1 mmol) in anhydrous CH_2_Cl_2_ (20 mL) under an argon atmosphere was added titanium tetraisopropoxide (1 mmol), and the reaction mixture was stirred at room temperature for 15 min, and then was cooled to −20 °C for 20 min. A solution of TBHP (2 mmol) in anhydrous CH_2_Cl_2_ (3.2 mL) was added slowly, the mixture was stirred for 8 to 12 h at room temperature, and then water (20 mL) was added. The mixture was stirred for another 1 h, the resulting gel was dissolved with ethyl acetate (30 mL × 2). After the solvent was removed under reduced pressure, the crude oil was purified by columnchromatography.

*tert-Butyl (4-((5-phenyl-1H-tetrazol-1-yl)sulfonyl)butyl)carbamate* (**11d**): yield 73%, yellow soild; ^1^H-NMR (CDCl_3_): δ 7.68–7.70 (m, 2H), 7.63–7.58(m, 3H), 3.78 (t, *J* = 8.0 Hz, 2H), 3.18 (dd, *J*_1_ = 6.4 Hz, *J*_2_ = 12.4 Hz, 2H), 2.05–1.97 (m, 2H), 1.74–1.67 (m, 2H); ^13^C-NMR (CDCl_3_) δ 156.08, 153.53, 133.14, 131.60, 129.84, 125.21, 79.60, 55.68, 39.58, 28.80, 28.50, 19.64; MS: [M + H]^+^ 382.22; Mp: 94.1–95.5°C.

*tert-Butyl (4-(benzo[d]thiazol-2-ylsulfonyl)butyl)carbamate* (**11e**): yield 94%, yellow soild; ^1^H-NMR (CDCl_3_) δ 8.27–8.19 (m, 1H), 8.02 (d, *J* = 7.6 Hz, 1H), 7.67–7.55 (m, 2H), 3.55 (t, *J* = 8.0 Hz, 2H), 3.14 (d, *J* = 5.5 Hz, 2H), 1.97–1.89 (m, 2H), 1.66 (p, *J* = 7.1 Hz, 2H), 1.41 (s, 9H); ^13^C-NMR (CDCl_3_) δ 165.81, 156.01, 152.77, 136.83, 128.16, 127.78, 125.57, 122.45, 79.44, 54.34, 39.71, 28.84, 28.45, 19.84; MS: [M + H]^+^ 369.01; Mp: 103.6–104.5 °C.

### 3.9. General Procedure for the Synthesis of Sulfone Compounds ***9d*** and ***9e***

To a solution of compound **11** (1 mmol) in dry CH_2_Cl_2_ (10 mL) was added CF_3_COOH (2 mmol) and stirred at room temperature for 2 h. Then NaOH (1 mmol/L) was added until pH = 9, extracted with CH_2_Cl_2_ (10 mL). Then organic layer **12** was applied directly to the next step reaction followed as synthesis of compound **9**.

*1-((4-Isothiocyanatobutyl)sulfonyl)-5-phenyl-1H-tetrazole* (**9d**): yield 15%, yellow soild; ^1^H-NMR (CDCl_3_): δ 7.79–7.58 (m, 5H), 3.83 (t, *J* = 7.7 Hz, 2H), 3.64 (t, *J* = 6.4 Hz, 2H), 2.19–2.11 (m, 2H), 1.98–1.91 (m, 2H); ^13^C-NMR (CDCl_3_) δ 153.4, 133.0, 131.7, 129.9, 125.2, 55.2, 44.5, 28.5, 19.9; HRMS-ESI (+) for C_12_H_14_N_5_O_2_S_2_, calculated 324.0589, found 324.0613 [M + H]^+^; Mp: 91.4–92.7 °C.

*2-((4-Isothiocyanatobutyl)sulfonyl)benzo[d]thiazole* (**9e**): yield 22%, yellow soild; ^1^H-NMR (CDCl_3_) δ 8.24 (d, *J* = 8.0 Hz, 1H), 8.04 (d, *J* = 7.6 Hz, 1H), 7.60–7.68 (m, 2H), 3.56–3.60 (m, 4H), 2.09–2.02 (m, 2H), 1.94–1.87 (m, 2H); ^13^C-NMR (CDCl_3_) δ 165.5, 152.8, 136.9, 128.3, 127.9, 125.7, 122.5, 53.8, 44.5, 28.6, 20.1; HRMS-ESI (+) for C_12_H_12_N_2_O_2_S_3_Na, calculated 334.9958, found 334.9978 [M + Na]^+^; Mp: 101.1–102.6 °C.

### 3.10. General Procedure for the Synthesis of Tetrazole Acids Compounds ***14a***–***b***

To a solution of compound **3d** (1 mmol) in dried CH_2_Cl_2_ (2 mL) was added DMAP (0.2 mmol) and stirred at room temperature for 5 min. Then N-acetylcysteine (0.8 mmol) or thiopronin (0.8 mmol) was added, and the reaction mixture was stirred for 4 h. Added 10% aqueous citric acid solution (10 mL) and CH_2_Cl_2_ (10 mL). Then organic layer was dried with anhydrous sodium sulfate, then purified by silica gel column and got compound **14**.

*2-(((4-((5-Phenyl-1H-tetrazol-1-yl)thio)butyl)carbamothioyl)thio)acetic acid* (**14a**): yield 16%, yellow oil; ^1^H-NMR (CDCl_3_): δ 7.57–7.55 (m, 5H), 4.04 (q, *J*_1_ = 8.5 Hz, *J*_2_ = 6.9 Hz, 2H), 4.00 (s, 2H), 3.41 (q, *J*_1_ = 8.3 Hz, *J*_2_ = 7.5 Hz, 2H), 2.04–7.73 (m, 4H); ^13^C-NMR (CDCl_3_) δ 201.4, 174.0, 154.2, 133.6, 130.2, 129.8, 123.9, 43.8, 35.5, 32.6, 27.0, 26.3. HRMS-ESI (+) for C_1_4H_18_N_5_O_2_S_3_, calculated 384.0623, found 384.0625 [M + H]^+^.

*2-(2-(((4-((5-Phenyl-1H-tetrazol-1-yl)thio)butyl)carbamothioyl)thio)propanamido)acetic acid* (**14b**): yield 23%, yellow oil; ^1^H-NMR (CDCl_3_): δ 8.75 (t, *J* = 5.2 Hz, 1H), 7.57–7.56 (m, 5H), 7.44 (t, *J* = 5.6 Hz, 1H), 4.55 (q, *J* = 7.2 Hz, 1H), 4.15 (dd, *J*_1_ = 6.0 Hz, *J*_2_ = 18.0 Hz, 1H), 3.97 (dd, *J*_1_ = 5.2 Hz, *J*_2_ = 18.4 Hz, 1H), 3.91–3.83 (m, 1H), 3.80–3.72 (m, 1H), 3.44–3.34 (m, 2H), 1.97–1.91 (m, 2H), 1.88–1.83 (m, 2H), 1.55 (d, *J* = 7.6 Hz, 3H); ^13^C-NMR (CDCl_3_) δ 195.5, 177.2, 173.5, 154.6, 133.5, 130.3, 129.9, 124.0, 46.7, 32.7, 29.8, 27.0, 26.7, 20.9, 16.5; HRMS-ESI (+) for C_17_H_23_N_6_O_3_S_3_, calculated 455.0994, found 455.0995 [M + H]^+^.

### 3.11. General Procedure for the Synthesis Tetrazole N-dimethyl Compound ***14c***

To a solution of 2-(dimethylamino)ethanethiol (0.71 mmol) in 95% EtOH (1 mL) was added **3d** (0.71 mmol), and the reaction mixture was stirred for 10 h at 40 °C. Then organic layer was dried with anhydrous sodium sulfate, then purified by silica gel column to give compound **14c**.

*2-(Dimethylamino)ethyl(4-((5-phenyl-1H-tetrazol-1-yl)thio)butyl)carbamodithioate* (**14c**): yield 20%, yellow oil; ^1^H-NMR (CDCl_3_): δ 11.07 (s, 1H), δ 7.55–7.51 (m, 5H), 3.71–3.66 (m, 2H), 3.39 (t, *J* = 7.2 Hz, 2H), 3.01 (t, *J* = 5.2 Hz, 2H), 2.72 (t, *J* = 5.6 Hz, 2H). 2.30 (s, 6H), 1.96–1.84 (m, 2H), 1.82–1.75 (m, 2H); ^13^C-NMR (CDCl_3_) δ 196.4, 154.2, 133.5, 130.1, 129.8, 123.8, 60.9, 46.2, 45.1, 34.0, 32.6, 27.2, 26.7; HRMS-ESI (+) for C_16_H_25_N_6_S_3_, calculated 397.1303, found 397.1298 [M + H]^+^.

### 3.12. Bioogical Activity

#### 3.12.1. Inhibition of Cell Growth *in Vitro*

MCF-7, SUM-159, KG-1a and 293T cellswere from GuangzhouJennio Biotech Co.Ltd. (Guangzhou, China). They were cultured in RPMI 1640 supplemented with 10% FBS at 37 °C in a 5% CO_2_ incubator. Cells were seeded in 96-well plates at a density of 3000 cells per well. All cells were treated for 48 h, with increasing concentrations of different compounds.Cell viability was measured using an MTT assay which was performed following the manufacturer’s protocol. The number of living cells was directly proportional to the absorbance at 490 nm of a formazan product reduced from MTT by living cells. The IC_50_ value was obtained using SPSS 11.5 software. The results were derived from three independent experiments performed in triplicate.

#### 3.12.2. Caspase-3 Activity Assay

SUM-159 cells were treated with SFN (12 µM), **3d** (4.5 µM), **14c** (9.5 µM), respectively, and collected after 48 h. The caspase-3 activity assay was determined using a caspase-3 activity kit (Beyotime, Nanjing, China). Cellular protein was extracted with the supplied lysis buffer, followed by the determination of protein concentration using BCA Protein Assay Reagents (Beyotime). The assay is based on the ability of caspase-3 to change acetyl-Asp-Glu-Val-Asp *p*-nitroanilide into the yellow formazan product, *p*-nitroaniline. The absorbance at 405 nm was determined, and the activity of caspase-3 was assessed by calculating the ratio at OD_405nm_ of the drug-treated cells to the untreated cells. The results were derived from three independent experiments performed in triplicate.

#### 3.12.3. Aldefluor Assay

A cell population with a high aldehyde dehydrogenase (ALDH) enzyme activity was previously reported to enrich mammary stem/progenitor cells. SUM-159 cells were treated with SFN (1 and 5 µM), **3d** (0.5 and 2.5 µM), **14c** (0.5 and 2.5 µM) and DMSO for 4 days and subject to Aldefluor assay and flow cytometry analysis. (Stem Cell Technologies, BD, New York, NY, USA). Single cells obtained from the drug-treated cells were incubated in an Aldefluor assay buffer containing an ALDH substrate, bodipy-aminoacetaldehyde (1 µM per 1,000,000 cells), for 40 to 50 min at 37 °C. As a negative control, a fraction of cells from each sample was incubated under identical condition in the presence of the ALDH inhibitor diethylaminobenzaldehyde. Flow cytometry was used to measure ALDH-positive cell population. The results were derived from three independent experiments performed in triplicate.

## 4. Conclusions

The ALDH^+^ population in breast cancer cell line SUM-159 was stem cell-like cells, and it was recently reported that SFN can selectively inhibit the growth of this cell population [8]. To further investigate the structure-activity relationship of SFN, a series of SFN analogues with heterocyclic moieties were synthesized in this study, and then were assayed against the breast cancer cell lines, MCF-7 and SUM-159, and the acute leukemia stem-cell like cell line KG-1a. Among the furan, methoxypyridine, methoxybenzene, tetrazole, and thiazole classes of SFN analogues, the tetrazole SFN analogues **3d**, **8d**, and **9d** were generally the most potent. In particular, **8d** exhibited up to a 15-fold greater potency than SFN against KG-1a cell line. The water soluble derivatives **14c** exhibited comparable activity with the parent molecule, **3d**, against MCF-7 and SUM-159, and higher potency against the KG-1a cell line. In addition, compounds **14c** exhibited less effect than **3d** on the human embryonic kidney cell line 293T.Caspase-3 activity data also indicated that SFN, **3d** and **14c** induced apoptosis in the SUM-159 cell line by increasing caspase-3 activity. Like SFN, analogues **3d** and **14c** also significantly reduced the ALDH^+^ subpopulation in the SUM-159 cell line from 3.10% to 0.16% and 0.07%, respectively. In contrast, treatment with DOX increased the ALDH^+^ population to 5.12%. Based on the results obtained for the leukemia stem-cell like cell line KG-1a, and the observed effects on the stem cell-like ALDH^+^ subpopulation in breast cancer cells, the biological activities of **3d** and **14c** should be further investigated.

## Figures and Tables

**Table 1:** Inhibitory effects of SFN analogues **3a**–**e**, **8a**–**e**, **9a**–**e** on MCF-7, SUM-159 and KG-1a cell lines.
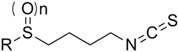

Entry	Compound	R	n	IC_50_ (µM)		
				MCF-7	SUM-159	KG1-a
1	SFN	methyl	2	24.11 ± 6.62	7.69 ± 0.92	8.24 ± 2.81
2	**3a**	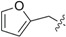	0	69.31 ± 11.45	>33.33	15.28 ± 2.91
3	**8a**		1	47.20 ± 5.42	>33.33	10.87 ± 3.14
4	**9a**		2	23.30 ± 4.67	>33.33	11.98 ± 1.46
5	**3b**	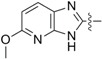	0	16.26 ± 1.27	5.94 ± 1.74	10.07 ± 1.07
6	**8b**		1	27.72 ± 2.96	>33.3	25.76 ± 5.08
7	**9b**		2	32.91 ± 2.23	>33.3	73.1 ± 3.08
8	**3c**	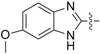	0	9.13 ± 0.99	5.34 ± 0.30	6.30 ± 1.45
9	**8c**		1	8.15 ± 2.49	1.45 ± 0.47	4.01 ± 0.97
10	**9c**		2	26.78 ± 2.7	5.88 ± 0.96	10.95 ± 2.38
11	**3d**	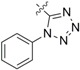	0	2.66 ± 0.25	1.46 ± 0.19	1.52 ± 0.38
12	**8d**		1	4.11 ± 0.9	1.54 ± 0.29	0.51 ± 0.14
13	**9d**		2	1.66 ± 0.23	2.08 ± 0.24	0.88 ± 0.28
14	**3e**	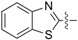	0	8.04 ± 1.86	>33.33	14.39 ± 3.69
15	**8e**		1	16.30 ± 2.65	3.94 ± 0.88	5.18 ± 0.57
16	**9e**		2	31.50 ± 4.2	>33.33	6.12 ± 0.97

**Table 2:** Inhibitory effects (IC_50_) of water soluble compounds **14a**–**c** on MCF-7, SUM-159 and KG-1a cell lines.
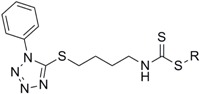

Entry	Compound	R	IC_50_ (µM)		
			MCF-7	SUM-159	KG-1a
1	SFN	—	15.39 ± 2.44	10.61 ± 0.91	11.45 ± 1.37
2	**3d**	—	4.33 ± 0.41	4.90 ± 0.57	3.40 ± 0.08
3	**14a**	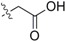	13.66 ± 1.47	29.05 ± 0.66	17.54 ± 2.2
4	**14b**	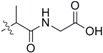	7.84 ± 1.20	5.96 ± 0.29	4.73 ± 0.22
5	**14c**	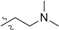	7.30 ± 1.35	6.71 ± 1.47	1.88 ± 0.44

**Table 3:** Effect of compounds **14a**–**c** on 293T cell.

Compound	CC_50_ (μM)
**SFN**	7.84 ± 0.72
**3d**	2.12 ± 0.15
**14a**	10.78 ± 0.80
**14b**	5.39 ± 1.03
**14c**	4.56 ± 1.26
